# Potential of deep learning segmentation for the extraction of archaeological features from historical map series

**DOI:** 10.1002/arp.1807

**Published:** 2021-01-26

**Authors:** Arnau Garcia‐Molsosa, Hector A. Orengo, Dan Lawrence, Graham Philip, Kristen Hopper, Cameron A. Petrie

**Affiliations:** ^1^ Landscape Archaeology Research Group (GIAP) Catalan Institute of Classical Archaeology Pl. Rovellat s/n Tarragona 43003 Spain; ^2^ Department of Archaeology Durham University South Road Durham DH1 3LE UK; ^3^ Department of Archaeology University of Cambridge Downing Street Cambridge CB2 3DZ UK

## Abstract

Historical maps present a unique depiction of past landscapes, providing evidence for a wide range of information such as settlement distribution, past land use, natural resources, transport networks, toponymy and other natural and cultural data within an explicitly spatial context. Maps produced before the expansion of large‐scale mechanized agriculture reflect a landscape that is lost today. Of particular interest to us is the great quantity of archaeologically relevant information that these maps recorded, both deliberately and incidentally. Despite the importance of the information they contain, researchers have only recently begun to automatically digitize and extract data from such maps as coherent information, rather than manually examine a raster image. However, these new approaches have focused on specific types of information that cannot be used directly for archaeological or heritage purposes. This paper provides a proof of concept of the application of deep learning techniques to extract archaeological information from historical maps in an automated manner. Early twentieth century colonial map series have been chosen, as they provide enough time depth to avoid many recent large‐scale landscape modifications and cover very large areas (comprising several countries). The use of common symbology and conventions enhance the applicability of the method. The results show deep learning to be an efficient tool for the recovery of georeferenced, archaeologically relevant information that is represented as conventional signs, line‐drawings and text in historical maps. The method can provide excellent results when an adequate training dataset has been gathered and is therefore at its best when applied to the large map series that can supply such information. The deep learning approaches described here open up the possibility to map sites and features across entire map series much more quickly and coherently than other available methods, opening up the potential to reconstruct archaeological landscapes at continental scales.

## 1 INTRODUCTION

The use of historical maps has a long tradition in archaeological research and has played an important role in a wide array of scientific disciplines (Chiang, Duan, Leyk, Uhl, & Knoblock, 2020). As a ‘frozen’ image of a territory, maps provide information about the landscape and society of the period in which they were created. Beyond that, they are also useful for analysing aspects of the landscape which have since been truncated or destroyed by the types of large‐scale transformations conducted over the last century, in particular, mechanized agriculture and urban expansion. Archaeologists have been using these sources for a long time in order to carry out regressive analysis and reconstruct successive landscape phases through time employing old maps for the study of historical patterns in settlement, road networks and field systems (Bevan & Conolly, 2002; Chouquer, 1996; Crawford, 1926; Hoskins, 1955; Orengo & Palet, 2009; Vermeulen, Antrop, Hageman, & Wiedemann, 2001; Vion, 1989). Historical maps can also be used to identify archaeological sites or features which were reported, on purpose or accidentally, by the surveyors (Lape, 2002; Orengo & Fiz, 2008; Panich, Schneider, & Byram, 2018; Petrie et al., 2019; Rondelli, Stride, & García‐Granero, 2013).

Systematic survey and mapping have been an essential and widely used instrument of statecraft for centuries, used to conquer, control, manage, tax, exploit, divide and protect areas. Since the late eighteenth century, the development of survey techniques on one side and political and ideological interests on the other pushed several European states to undertake systematic mapping of their own territories at an unprecedented scale and extension (Kent, Vervust, Demhardt, & Millea, 2020). This step change in European map production was almost immediately applied in their colonial dominions, starting during the nineteenth century, thereby reaching large parts of the world, as an inseparable companion of enlightenment, imperialism, agricultural intensification and the industrial revolution. In the aftermath of the First World War, imperial dominions extended through large parts of the Middle East and marked the beginning in the use of aerial survey techniques for large scale mapping.

The Cassini Carte of France, the British Ordnance Survey and the Russian mapping of Siberia and Central Asia are examples of grand projects that are well known and employed within archaeological research. In this context can be placed the two series used in this work: the Survey of India (SoI), which was initially developed in parallel with the expansion of British control in India during the nineteenth century (Edney, 2009; Sarkar, 2020), and the 1:50.000 series derived from the works performed by the Bureau Topographique du Levant (BTL, later renamed Service Géographique des Forces Française Libres du Levant) created in 1918 under the authority of the Service Géographique de l'Armée (Le Douarin, 2020). Despite significant differences in technical apparatus, many of these maps were produced to a very high standard, with a spatial accuracy which is almost comparable to that of modern maps at similar scales.

The vast amount of information resulting from the continuous systematic mapping projects conducted between the late eighteenth century and the middle of the twentieth century remains in physical archives. Many institutions are currently developing digitizing programs to make these maps more readily available. Maps have been digitized on demand for research purposes, and digital repositories are becoming available. However, the number of digital historical maps is still relatively small in comparison to the total coverage, and to collect the maps necessary to ensure coverage of a large study area usually requires access to several repositories and the digitization of archive‐stored originals often hosted in multiple institutions.

These map series offer considerable potential for archaeological and historical research and also heritage protection and management as they often record archaeological sites, historical monuments and other features of archaeological interest, either deliberately or incidentally through features such as place names, specific symbology or topographic expressions (Petrie et al., 2019). These colonial map series were produced intensively during the nineteenth and early twentieth centuries, and they depict landscapes that have been substantially modified since their production. During the last half century, the adoption of mechanized agriculture, intensive irrigation, urban development and in some areas conflict and large‐scale looting has dramatically changed the landscapes reflected in these maps, making them much more valuable for archaeologists and historians. In many cases, they include archaeological features which are difficult to identify today and may be entirely destroyed. Associated information such as toponyms is also very valuable, as they document historic knowledge that might also be lost today. In many regions, the quality and importance of the information contained in these map series should qualify them as one of the basic, most relevant sources for archaeological, historical and heritage research. However, this has rarely been the case, and, although many projects make use of these historical maps, there have been few systematic attempts to extract information as large‐scale quantifiable georeferenced data.

Up to now, most uses of historical map collections have relied on the digitization of maps as raster image files and their more or less systematic georeferencing in GIS environments (Orengo, Krahtopoulou, Garcia‐Molsosa, Palaiochoritis, & Stamati, 2015; Petrie et al., 2019). However, the most time‐consuming part, the extraction of features of historic‐archaeological interest, has had to be done using manual approaches. This process has involved the visual identification/location of features and their digitization using vector formats that could correspond to points (the fastest of the methods), lines or polygons (which provide extra information such as shape and area but require a higher investment of labour). There has been a recent increase in the development of approaches directed to the automatic vectorization of maps (Chiang et al., 2020; Shbita et al., 2020; Uhl, Leyk, Chiang, Duan, & Knoblock, 2020). Those cases take advantage of current developments in machine learning (ML) and deep learning (DL) approaches to computer vision (CV), with neural networks (NNs) having a prominent role. These approaches largely remain experimental and complex and do not categorize elements of archaeological interest. Notably, the archaeologically relevant information is included within other categories of data such as topography, toponymy or specific map symbology and still requires manual extraction and analysis.

**FIGURE 1:**
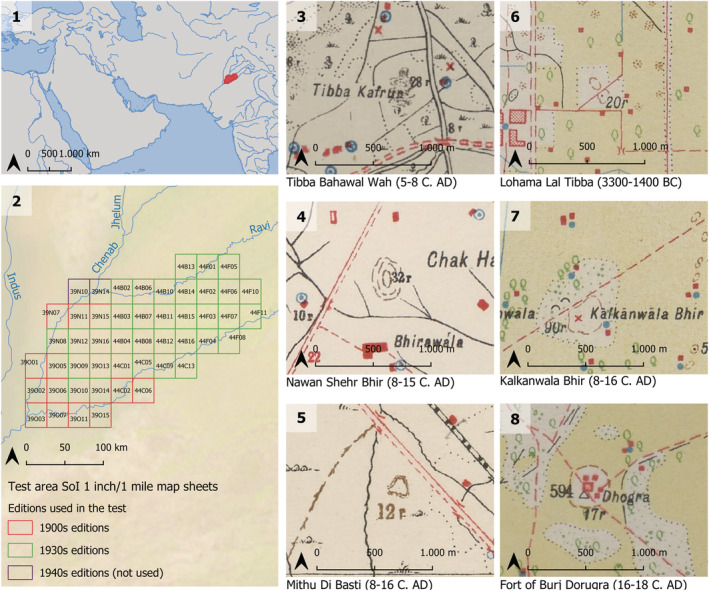
(1) Location of the area used for testing the survey of India collections and (2) sheets used in this paper. Examples of archaeological sites (locations obtained from Mughal, Khan, Iqbal, Hassan, & Afzal Khan, 1996) drawn as mounds in pre‐WWI editions (3–5) and interwars editions (6–8) [Colour figure can be viewed at wileyonlinelibrary.com]

**FIGURE 2:**
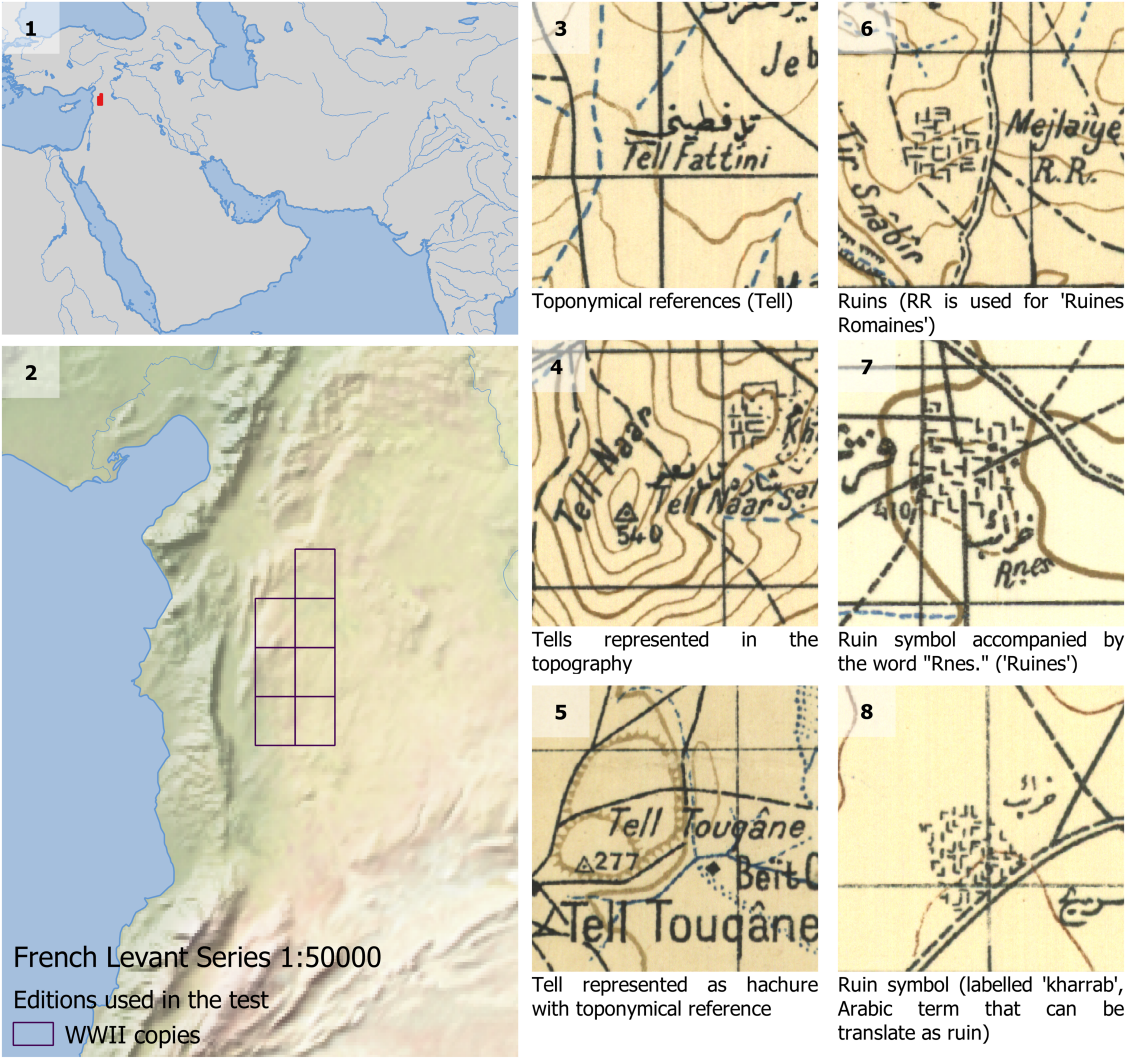
(1 and 2) location of the area of Syria covered by the maps used in this test. Different examples to represent potential archaeological mounds (3–5) and the presence of settlement ruins (6–8). Note that ‘tell’ (Arabic for settlement mound) may appear as the name of a mound feature or a toponym in the absence of an obvious topographic feature. This convention may be due to the placement of the names on the map, or a real difference in the location of the named village and the tell site, or because the tell has been destroyed in advance of the mapping of the region [Colour figure can be viewed at wileyonlinelibrary.com]

In this paper, we provide a first proof of concept for the automatic extraction of features of archaeological interest from large series of historical maps using DL approaches. For this purpose, we have selected two map series depicting areas of high archaeological potential that were produced by two different colonial governments: the SoI during the period of British control of South Asia (Figure 1) and the ‘Armée Du Levant. Service Géographique’ series during the period of the French Mandate in Syria and Lebanon (Figure 2). For the latter, we have used a series of copies made during World War 2 and its aftermath, as these were readily available. The projection system and information depicted are the same in the copies, though they have a narrower range of colours than the original series. These map series offer enough variability to test the identification and extraction of different types of features with different levels of detection complexity and archaeological interest.

## 2 METHODS: DL‐BASED SEGMENTATION

ML, a subfield of artificial intelligence, has only recently begun to be exploited in archaeological research, but applications are rapidly increasing. The field developed significantly during the second half of the 2010s when archaeologists started to take advantage of easy access to ML algorithms and cloud computing resources. Most of these applications have focused on the detection of archaeological sites, usually employing remote sensing data (Davis, 2020). They include examples of the use of LiDAR (Davis, Lipo, & Sanger, 2019; Gallwey, Eyre, Tonkins, & Coggan, 2019; Somrak, Džeroski, & Kokalj, 2020; Trier, Cowley, & Waldeland, 2019; Verschoof‐van der Vaart, Lambers, Kowalczyk, & Bourgeois, 2020), multispectral satellite imagery (Menze & Ur, 2012; Orengo et al., 2020; Thabeng, Merlo, & Adam, 2019; Trier, Larsen, & Solberg, 2009) and/or drone‐based imagery (Orengo & Garcia‐Molsosa, 2019; Sărășan et al., 2020). Successful approaches have been tested, but the use of these technologies is still very limited outside research groups dedicated to experimentation on computational applications in archaeology. ML approaches require computational skills that are not routinely taught in archaeological training. Where archaeologists have taken the plunge, the complex alignment of sources, technical capacities and research questions required can produce disappointing outcomes. As a result, there is some understandable scepticism towards its practical utility (Casana, 2014; Palmer, 2020).

Despite their potential, historical maps have been left outside this approach. This is likely due to several factors:

1. A certain amount of preprocessing is necessary to apply ML methods, such as digitization and georeferencing.
2. Maps are not always easy to access, and there are few complete historical map series that can be freely accessed and downloaded in digital form.
3. Maps are subjective sources made by surveyors whose interest was rarely the recording of archaeological sites. As such, they often lack strict, systematic parameters that can be used to identify archaeological sites. Sites can be represented through its topography but also conventional signs, both intended and unintended. That means that the same type of cultural element might be represented by different symbology in the same collection or even the same map. Also, the inclusion or not of a site mark in a map is highly dependent on the surveyor perception.
4. In contrast with current DL archaeological applications, which usually focus on simple shapes such as mounds visible in lidar‐derived topographic data, features of interest in historical maps, even within the same object class, present inconsistent and irregular shapes. Their detection requires a much larger quantity of training data and the use of data augmentation techniques.

The first two factors that have influenced the use of historical maps can be at least partially overcome by choosing appropriate map series and working across multiple institutions. We might also expect accessibility of map series to increase over time as more institutions digitize their collections. Importantly, the age of many of the map series means they are no longer subject to copyright and it is possible to make them publicly available with limited restrictions on reuse. The last two factors are more challenging to overcome, but we believe that a systematic extraction of archaeological and heritage features from historical maps is not just possible but beneficial under certain circumstances.

It is important not to overlook the fact that many of the identified features need to be verified in the field. This presents additional problems because landscape change and inaccuracies in the recording, georeferencing and placement of tags can make the ground checking of map‐recorded features complicated.

Our approach to implementing ML on historical maps is based on two separate steps.

### 2.1 Georeferencing of high‐resolution digitized historical maps

For the SoI map series, a detailed description of the georeferencing procedures that have been developed and implemented can be found in Petrie et al. (2019) and Green et al. (2019). A short summary is offered here.

Both ESRI's ArcMap (ESRI, 2020) and QGIS georeferencing tools (a plugin using GDAL in the case of QGIS; QGIS, 2020) were employed for the georeferencing process using WGS84 as the geodetic datum. Ground control points (GCPs) were obtained in ArcGIS through its basemap service and QGIS using high‐resolution Bing and Google imagery services imported as XYZ tiles.

Because the maps were digitized using either a photographic camera or a barrel scanner and their preservation state was not ideal, we employed a minimum of 20 clearly identifiable GCPs distributed evenly across each map. These consisted mostly of canal, road, and railroad intersections, which were some of the few landscape elements that have been preserved since the early twentieth century. GCPs for each map were evaluated using their RMSE values, and unreliable GCPs were eliminated to achieve the best possible result. The rectified maps provided maximum RMSE values of 26.8 m.

The French mandate Levant series were georeferenced using ESRI's ArcMap georeferencing tool. Eight figure grid references on the French‐British Levant Lambert projection grid (equivalent to the modern Deir ez Zor/Syrian Lambert system which uses the Deir ez Zor geographic 2D CRS) and the Syria Lambert (Lambert Conic Conformal 1SP as its projection) are printed in the corner of each map sheet, giving a precision to the nearest 100 m. These were used as control points. Rectified maps provided maximum RMSE values of 65.5 m. For comparison with other datasets, the maps were then reprojected in the WGS84 UTM coordinate system.

### 2.2 CNN‐based DL segmentation of features of interest in digitized historical maps

Although mounded shapes characterize many of the archaeological sites in our two test areas, these features do not follow a unique or standard form of representation. Mounds and other features of interest can be represented using a variety of symbols and toponymy (see Petrie et al., 2019). In this work, we test three different types of representation of features of archaeological interest: topographic elements to represent mounds, complex conventional signs for the representation of ‘ruins’ and toponyms linked to archaeological features. The first is tested on the SoI collection (Figure 1: 3–8) and the latter two on the Levant series (Figure 2: 6–8 for conventional signs and 3–5 for toponyms).

The strategy adopted here makes use of segmentation approaches as, besides site location, we were interested in the shape and size of the features of interest, in particular mound representations. Rather than employing a single detector to classify the whole map series, we developed different detectors focusing on specific elements of interest. This strategy allowed us to have a more focused training process in which only a particular element per detector was tagged, avoiding confusion between classes.

Given the number of classifiers required to detect all objects of archaeological interest, we employed Picterra, an online ML platform that provides a simple and intuitive graphical interface for the selection of training data. Picterra uses a U‐Net‐based architecture (Ronneberger, Fischer, & Brox, 2015) for the ML object instance segmentation. Convolutional neural networks (CNN) are DL architectures that, among other uses, can identify and outline predefined objects classes from raster images through the patterns in pixel relations. This approach is well suited for identifying individual objects not necessarily identical but that share a similar representation on the maps.

Typical DL methods combine object detection to classify individual objects and locate each of these within a bounding box, and semantic segmentation, which classifies each image pixel into a category and instance segmentation, in order to differentiate between object instances. Picterra implementation uses a CNN architecture based on U‐Net. The algorithm automatically performs a series of preprocessing steps including data augmentation, which aims at providing well‐balanced and effective training data for the development of the model. The training of the models uses cloud‐based distributed computing, which greatly speeds the process. In addition, by using proprietary postprocessing techniques on the output of the U‐Net model, it separates the per‐pixel classification results into separate objects, effectively outputting Mask R‐CNN‐like instance segmentation results without the overhead of a large and overly complex network that requires an abundance of data to be trained on. In this way, the training and testing of detectors can be achieved very quickly without the need to gather large amounts of annotated images. There are two other reasons why Picterra was considered an adequate platform for this research instead of developing our own open access detectors: (a) the research aim was to test the potential of DL for the detection of multiple map features, and therefore, a fast and efficient method allowed us to experiment until an adequate detector for each feature was achieved; (b) the symbology and representation of archaeological features can vary greatly between series and between maps in individual series, meaning other researchers will have to train their own algorithm that fits the specific features and symbology of the maps they are using.

### 2.3 Map series and training of the algorithm

The maps were analysed by experts in the archaeology of each study area in order to select relevant features that were indicative of archaeological sites.

The historical series of the SoI is composed of hundreds of sheets and covers much of modern Pakistan, India, Bangladesh and Sri Lanka. The 1″ to 1‐mile series (1:63,360) of SoI maps is the most detailed scale commonly available, and detailed four‐colour sheets were issued between 1905 and 1936, with much of the data being based on late nineteenth and early twentieth century surveys (Petrie et al., 2019). Consistent guidelines for the representation of different features were followed, though variations can be found in the different published series, and sometimes in various maps of the same series. Although the SoI is a government institution, maps were made available to libraries and the public from the moment of their publication and copies circulated widely outside military circles. We have worked with the collection stored in the Map Room of the Cambridge University Library which, as a copyright library, received copies of most of the maps that reached Britain and now holds one of the more complete collections in the United Kingdom. The British Library and the Bodleian Library also hold substantial collections of the 1″ to 1‐mile series, and although there is much overlap, these collections also complement each other. The US Army produced copies of the SoI maps, and these copies have been digitized by the University of Texas (US Army Service, 1955), which has made them publicly available.

**FIGURE 3:**
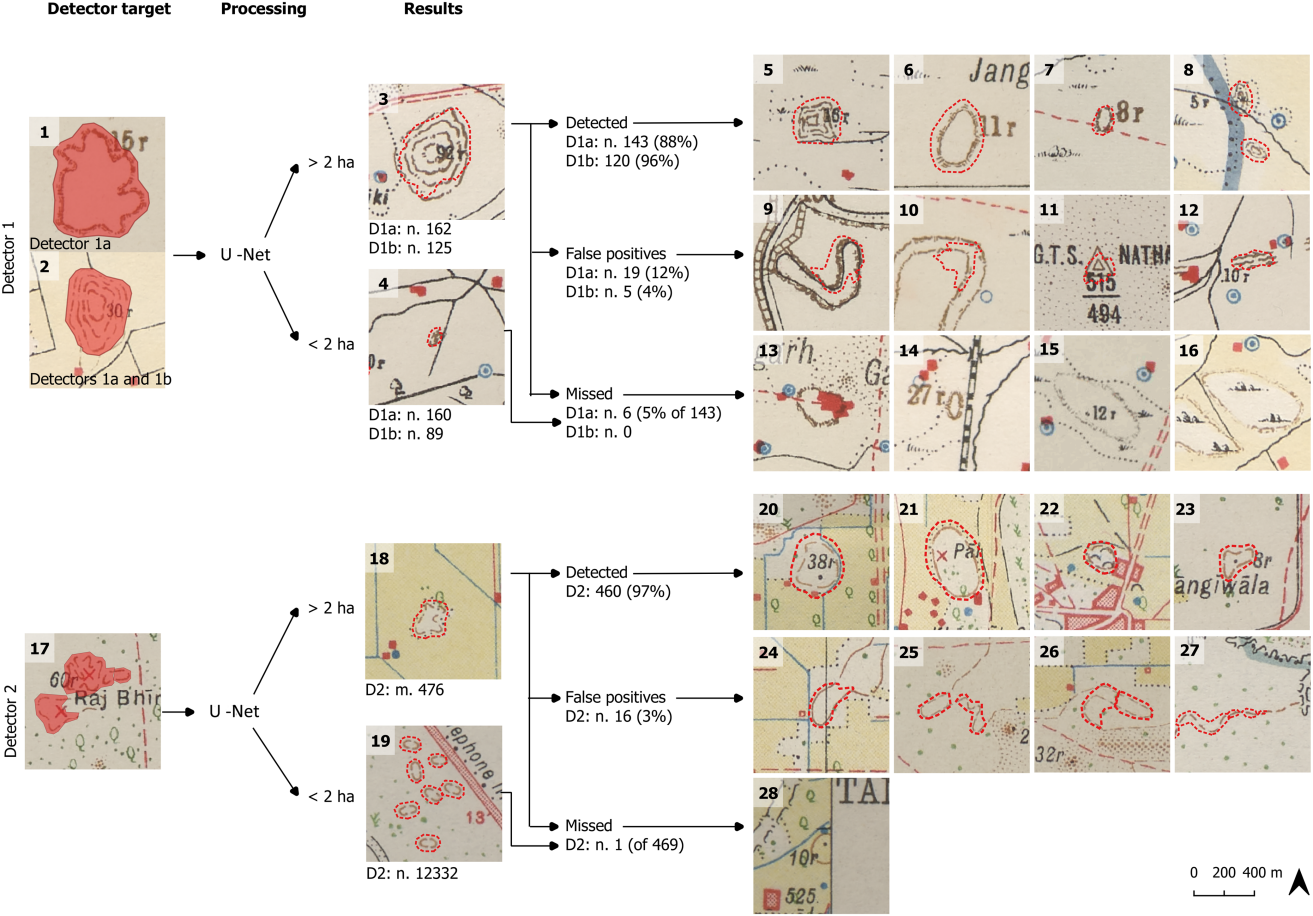
Detectors trained for the mounds represented at the (1–2) early 1900s and (17) 1930s editions of the SoI maps. The processing (3–4 and 18–19) included the use of a 2 ha threshold to discriminate the features with a higher probability of corresponding to archaeological sites. Examples of features successfully detected (5–8 and 20–23), false positives (9–12 and 24–27) and missed by the detector but manually detected (13–16 and 28) are illustrated [Colour figure can be viewed at wileyonlinelibrary.com]

In the case of the SoI maps, these have been previously employed to support archaeological survey in South Asia (Petrie et al., 2019) in particular in the Indian State of Haryana (Green et al., 2019) and the Pakistani Province of Punjab (Garcia, Orengo, Conesa, Green, & Petrie, 2019). These previous survey campaigns have been focused on mounds represented in the maps, which in many cases correspond to the remains of ancient settlements and also evidence for river courses and river migration. To some extent all the cultural phases that have seen settlement in the Indus River basin, from the time of the Indus civilization up to the British period, are represented in the rich archaeological record displayed in these maps. Field ground‐truthing in Haryana has confirmed the strong correlation between certain types of mound representations and the presence of surface archaeological material (Green et al., 2019). In a specific part of Haryana alone, 199 previously unknown archaeological sites have been detected and confirmed by ground assessment. This previous experience was important in selecting training data for the algorithm. Three different detectors were trained, each corresponding to a different type of mound representation (see Figure 3: 1–2 and 17). The test area corresponds to the current district of Multan in the Pakistani province of Punjab. The maps had been previously georeferenced, and the features extracted manually, providing the basis for testing of the results at a large scale.

The SoI maps used for this study (Figure 1: 2) correspond to two temporal series: 13 maps from the early 1900s editions, published between 1907 and 1909, and 34 maps from the 1930s editions, published between 1933 and 1936. In each edition, mounds are represented differently: in the 1900s editions, mounds typically appear as contours (Figure 1: 3 and 4) or hachures, a type of old topographical depiction similar to hillshading formed by roughly parallel lines, their closeness and density indicating steepness of gradient. They usually become thinner or display triangulated shapes as they point towards downhill direction (Figure 1: 5). In the 1930s editions, mounds are almost exclusively depicted as simple form lines (Figure 1: 6–8), though this type of representations may have also been used from the early 1910s (see Petrie et al., 2019). Thus, we have created a detector for each of the map series, as a way to obtain more accurate results:

- Detector 1a (Figure 3: 1 and 2) targets both ways of representing mounds in early 1900s maps: as contours and hachures. We have employed 26 training areas in eight of the maps, containing 71 features previously identified as mounds.
- Detector 1b (Figure 3: 1) is a second version of the early 1900s detector, which focuses only on contours. It was trained to test to what degree specialized detectors might improve the results within each map series. For this detector, we used 19 areas in five maps containing 66 features.
- Detector 2 (Figure 3: 17) targets simple discontinuous form lines and was applied to the 1930s maps. We employed 122 features recorded from 11 areas distributed in six maps.

A size‐based threshold was later applied to the results. In our experience in Haryana using SoI maps for field survey (Green et al., 2019), we have observed that features of a relatively larger size on the maps (from around 200 m diameter) are much more likely to correspond to archaeological sites confirmed by the presence of artefacts on the field. Conversely, smaller features (around 100 m or less in diameter; see example in Figure 3: 19) typically provided negative results during the fieldwork and likely indicated natural features, small dunes or modern spoil from pond excavation. As a result, we have divided the results into a high‐probability (>2 ha) category and a low‐probability (<2 ha) category and considered only the first group here. This threshold, however, presented a minor problem in the high‐probability group. In few cases, the detector identified larger features as several smaller features instead of a single larger one. The threshold, therefore, categorized these as low probability sites. On the contrary, groups of small features were often joined by the detector into larger single areas, which the threshold incorporated in the high‐probability range.

The case of the Levant map series of Syria is rather different. These were created during the 1930s, but most of the maps available, including those employed in this study, are British copies made during the Second World War. The Syria and Lebanon map series inherited models and practices from the North African French map series as their production was centralized by the Service Géographique de l'Armée, which dispatched the experienced officials who were in charge of the 1:50.000 series (Le Douarin, 2020). The French maps incorporate direct references to archaeological sites and some other representations that are indirectly related. The features/objects to be located included toponyms such as *tell* (mound in Arabic) and *khirba* (ruin in Arabic), which are indirectly related to archaeological sites, and also some text that can be directly associated with archaeological occurrences such as ‘R.R.’ (*Ruines Romaine*s), and ‘Ruines’ (ruins in French). The maps also include symbols marking ruins (Figure 2: 6–8) and some hachure symbology that can be related to mounded ‘tells’ (Figure 2: 5).

The maps of the French Levant 1:50,000 series have been used as a reference by archaeological surveys for many decades (Braemer, 1984, 1988). However, their use became widespread in archaeology only following easy digitization and accessible GIS‐software (Mantellini, Micale, & Peyronel, 2013).

**FIGURE 4:**
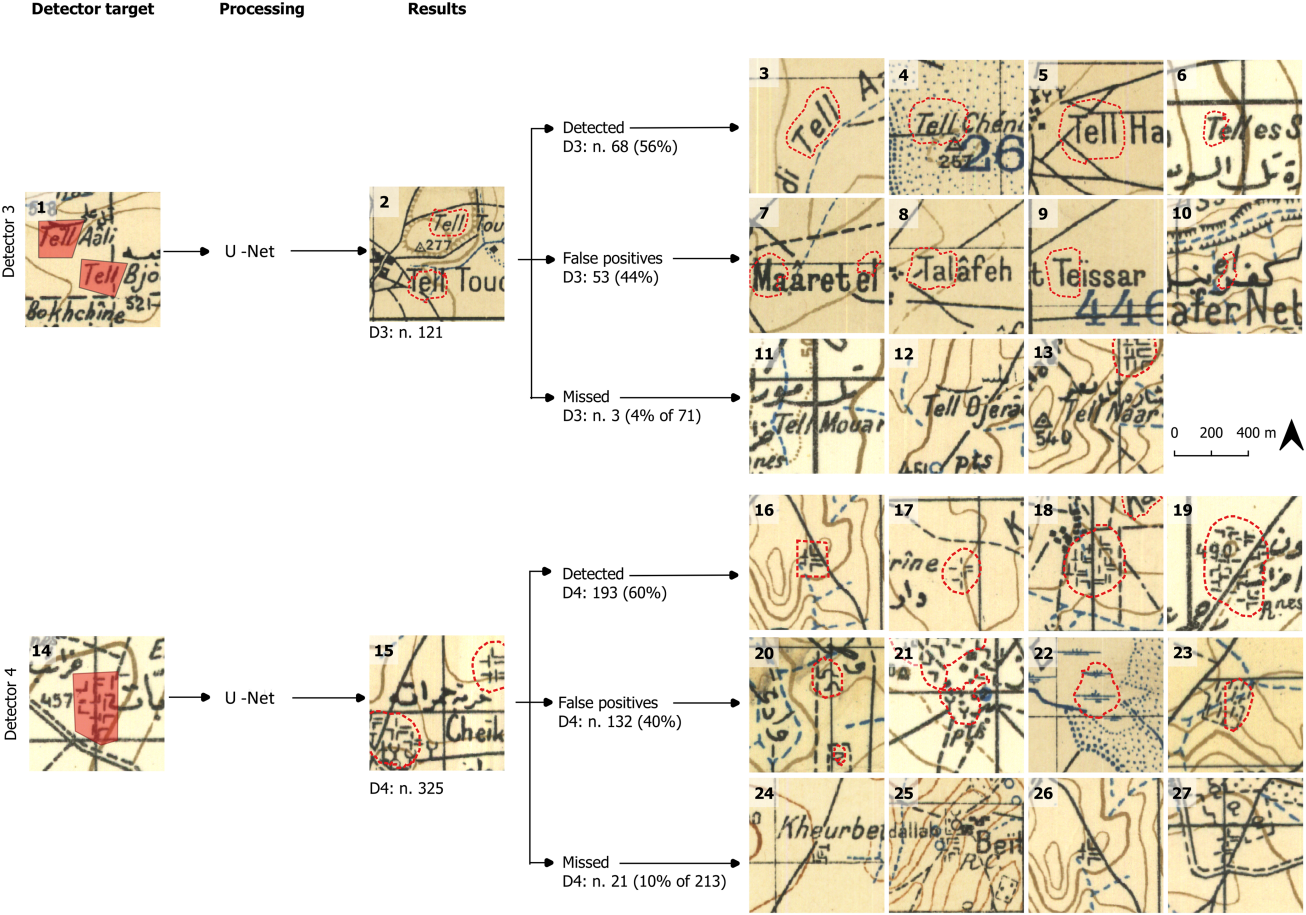
Detectors trained for the detection of ‘tell’ toponyms (1–2) and conventional sign for ruins (14–15) on French Levant maps. Examples of features successfully detected (3–6 and 16–19), false positives (7–10 and 20–23) and manually detected but missed by the detector (11–13 and 24–27) [Colour figure can be viewed at wileyonlinelibrary.com]

Two detectors developed for the Syria series have been trained on a small group of maps as a first test for the use of this methodology in the series:

- Detector 3 (Figure 4: 1) targeting the word ‘Tell’ in Latin characters was trained using eight training areas containing 39 features from three of the seven maps available for this preliminary work, as only three maps employed Latin characters. In the future, a similar detector for Arabic characters could be developed.
- Detector 4 (Figure 4: 14) focuses on the conventional symbol referring to ruins. It resembles a grouping of ‘L’‐shaped marks, perhaps indicating walls. Rather than using the single ‘L’ shape, which would have resulted in the detection of a large number of false positives given the simplicity of the symbol and its common appearance in other map features unrelated to ruins, the algorithm was trained using the ensemble of signs used to represent a single site. This is a complex type of symbolic representation. Although single symbols (such as red triangles) would have presented a much easier target, these composite symbols are challenging because of (1) the simplicity of the ‘L’ shape, which forms a part of many other symbols including letters and (2) the variable and changing way in which they are employed to represent sites. We used eight training areas from four different maps which contain 235 features in total.

## 3 RESULTS

Within the 47 maps of the SoI analysed, 13,130 features were identified through the DL process before using the size threshold, 322 features in the early 1900s maps and 12,808 in the 1930s maps. Applying the size threshold resulted in 638 high probability features (162 in the early 1900s maps and 476 in the 1930s maps).

Comparing the results with the systematic manual identification, all detectors employed have managed to detect at least 90% of the features identified through the manual identification (Table 1). In the case of the SoI, the detectors missed only seven features in total (Figure 3: 13–16 and 28), with the nuance that in a few cases the area identified is not large enough and, thus, once the threshold of 2 ha is applied the missing percentages increase to 10% in the early 1900a detectors tested (D1a and D1b) and 6% in D2.

**TABLE 1:** Summary of the accuracy obtained by the different detectors (see also Figures 3 and 4)

Automatic detected features				Manual identification	
Detector/feature type	Number of maps used	Features detected	False positives	Features detected^a^	Missed
D1a/mounds (SoI)	13	162^b^	12% (n. 19)	143	5% (n. 6)
D1b/mounds (SoI)		125^b^	4% (n. 5)	120	n. 0
D2/mounds (SoI)	34	476^b^	3% (n. 16)	469	n. 1
D3/toponyms (Levant)	3	121	44% (n. 53)	71	4% (n. 3)
D4/complex symbols (Levant)	7	325	40% (n. 132)	213	10% (n. 21)

^a^ Manual identification does not correspond exactly to automated detected features plus missed features—variation ranges from 0 (D1b and D3) to 8 (D2) cases for several reasons: missed during the manual inspection, features close to each other joined by the detectors or small features that fall under the threshold.

^b^ The numbers given here for the SoI maps are the result of applying a 2 ha threshold (see Figure 3).

In the SoI maps, the number of false positives is relatively small. It is significantly higher in D1a (12%) compared to the other two (4% D1b and 3% D2). Because most of the false positives in early 1900s editions were related to the hachured and form‐line features (Figure 3: 9 and 10), using a specific detector (D1b) that only considers contours increased the effectiveness. The limited number of hachures used in the maps analysed here makes it impossible to effectively train an algorithm for this type of feature, at least until more maps are incorporated. It is important to note that 15 out of 16 false positives identified by Detector 2 are located in a specific area in which the surveyor employed form lines to represent other topographic elements as well (Figure 3: 25–27).

On the three Levant series maps used for the Tell toponym identification, the DL process identified 121 features. On the seven sheets used for the Ruins symbols, Detector 4 identified 325 features. This finding represents a considerable increase compared to the manual identification (71 ‘Tell’ toponyms in three maps and 213 Ruin symbols in seven maps). As in the SoI, the detectors missed some of the manually identified features. The performance of Detector 3 was better (4%) than Detector 4 (10%). The test dataset for the Levant map series was a shapefile, which included all these symbols that had been identified by a manual survey of these maps undertaken some years ago as part of the Vanishing Landscape of Syria project.

In the Levant series, false positives represent around 40% of the total features identified. In the analysed series, the same type of continuous black lines is used for the letters and the ruins symbol, but also many other types of feature. Even so, most false positives obtained by the toponym detector correspond to similar letters or combinations of letters. For the ruins, the different combinations of L‐shape lines are very simple, and as a result, they are similar to other features.

In terms of time invested, once the researcher is familiarized with the platform and the maps, the process of training and obtaining the results can be done in a single work‐day (8 h for the 53 maps analysed here). Manually assessing this dataset would represent several weeks of work for an experienced operator. Increasing the number of maps would further reduce the time dedicated per map. The preprocessing of the maps, which include georeferencing and the assessment of the type of features, represented the most significant amount of time in the processing stage of the work.

## 4 DISCUSSION

The CNN‐based automated detection and instance segmentation method presented here is able to produce a reliable approximation of mounds and other features in both the SoI and Levant map series. These processes allow the production of digitized and geolocated areas of archaeological interest that can be used in the design of ground truth survey strategies and cultural heritage protection. It constitutes a quick and effective approach to develop preliminary information and initial hypotheses on the location, distribution and patterning of archaeological sites over large areas. These results could be combined with the analysis of remote sensing datasets to provide further support for interpretations made from map sources. Ultimately, however, field validation is still needed to confirm that a location is of archaeological interest.

Compared with the manual approaches commonly used, the automated detection results for the SoI maps are particularly effective in the identification of large mounds, which are strongly associated with archaeological sites. Of the 135 known archaeological sites depicted as mounds within the study area (Mughal et al., 1996), all but three were identified by the algorithm. Nonetheless, it has a less discriminant and interpretative capability than detailed human visual inspection. That limitation adds more noise to the dataset due to the existence of other types of small roughly circular features that are represented on the maps in a very similar way to settlement mounds, increasing the number of false positives. Size thresholds clearly have some potential for allowing us to overcome this problem but at the cost of missing some points of archaeological interest. A larger training dataset might help to identify better the shape of the features, which could increase the effectivity of the threshold.

However, the SoI‐focused detectors offer more coherent results than those resulting from manual extraction made by a group of analysts, particularly if they are not experienced or an effort has not been made to uniformize interpretations between team members.

The detectors tested in the Levant series have provided a first insight into two different types of archaeological information contained in historical maps: the toponymic reference and the conventional signs. Despite using a limited number of maps for the training, three for toponyms and four for complex site symbols, the results obtained show that the detectors missed relatively few of the targeted symbols and characters but did introduce a significant percentage of false positives. Thus, further work is still needed for these detectors to be employed effectively. The relatively small training dataset used for these maps and the much better performance provided by the SoI maps strongly suggests that the performance of the detectors can be significantly improved with a larger training dataset involving a more significant quantity of maps. Other editions of the Levant series, which incorporate colours and higher‐quality representations, could also provide better results than the Second World War editions of the Syria maps used in this first test.

Besides field validation, some strategies can be employed to analyse and interpret the data gathered. The use of other segmentation approaches in conjunction with remote sensing information on land use can help to filter data according to their landscape context. For example, in the Punjab study area, areas represented as barren land or active floodplains, which are less likely to contain archaeological mounds, can be automatically segmented from the maps after remote sensing validation. This step allows for a further assessment of the likelihood of the features identified being of archaeological interest and facilitates the development of thresholds to exclude lower levels of probability. The same approach could be applied to the Levant series maps, which represent different land uses patterns using hachures, a pattern which the detector occasionally mistakes for ruins signs. Besides obtaining information on land‐use history, these methods can be used to create masks that can help in reducing false positives in those maps as well.

Our results are provided as polygons rather than single points, providing information about the shape and area of the map features with much more detail than the point data typically produced using a visual assessment. The degree of accuracy is high, but some features are identified only partially, or small features close to one another are joined in a single feature. Much more accurate details could be obtained manually but would require a significant increase in time and labour. In any case, the relation of the feature on the map and the extension of the potential archaeological sites is merely indicative, because the number of factors involved makes a literal translation of the map features into real extents of archaeological sites on the ground extremely unlikely. This limitation is particularly true of the toponymic detection results.

The polygons enable the calculation of site areas, allowing, for example, experimentation on different levels of thresholds and degrees of probability and archaeological interest. They also facilitate the incorporation of these data in larger raster‐based geodatasets such as DEM‐derivates or vegetation indices (Orengo et al., 2019), which would be useful not just to validate features but also to assess the degree of preservation and to provide important training data to develop other types of ML‐based detection. Given the subjective nature of archaeological sites as depicted or indirectly reflected in these maps, the presence of false positives, and the possible disappearance of a part of these sites since their initial recording, it is worth considering this approach as part of a larger strategy. Geolocated detection results have enormous potential to be combined with not just ML detection from satellite data, but also existing site location data, traditional photointerpretation, survey, topographic analysis, crowd‐sourced locations and other site detection methods in a single probabilistic framework. A probabilistic approach would also give the opportunity to evaluate and cross‐validate the different sources and methods, alongside the information necessary to interpret their significance. This is particularly relevant when large areas are being studied, and expert‐led approaches or field validation are not possible or require major investment.

The method presents a significant scale factor. The larger the number of maps analysed and the quantity of features on each map, the more useful the approach tested in this study. The results obtained for the 1930s editions of the SoI, the only test in which we have employed a large number of maps, have proved particularly successful in terms of performance and time invested. That outcome suggests significant potential in scaling up the detectors to entire map series covering hundreds or thousands of individual sheets and across entire countries.

The use of computing platforms like Picterra provides a useful avenue for the implementation of automated detection to archaeological research. It is unreasonable to expect that all those archaeologists that could benefit from these approaches in academia, commercial archaeology or heritage management agencies will be able to build and train their own algorithms, especially given the computational capacity required. In that sense, the possibility to access ready‐made instruments and platforms can be beneficial in terms of testing different approaches and sources but also offers the chance to involve more traditional archaeologists in the development of their own detectors and help them understand the potential of the application of these technologies in our discipline. In that regard, automatic detection is in a position now to start making a practical contribution to the discipline and to be implemented as another instrument in the archaeologists' toolkit, in a similar way in which GIS was assimilated over the last 20 years (Wheatley & Gillings, 2013).

Applications of automated detection similar to the ones we have presented here have the potential to add significant value to the large collections of historical maps available on paper and in digital archives all over the world. Their potential for historical research in general is well known, but the information that they contain has been up to now hard to extract and quantify. Combining different ML techniques to speed up the vectorization process has genuinely transformative potential, particularly in large collections like the two analysed here. Just in this test study, we have identified 911 potential archaeological sites and have been able to assign probabilities relating to how far these are likely to correspond to actual sites. This is just a small sample of the potential data that can be extracted. The maps contain other information relevant for cultural heritage documentation, including forts, monuments, religious buildings and cemeteries. They are also some of the most important documents available for understanding environmental and landscape transformations during the early twentieth century, which were massive in the case of the areas of Haryana and Punjab (Agnihotri, 1996; Gilmartin, 2015) and Syria analysed here.

## 5 CONCLUSIONS

Automatic detection and instance segmentation of objects in digitized historical maps using ML CNN‐based approaches offer an efficient way forward for the retrieval of unique information of archaeological interest. However, the use of these approaches needs to take into account:

- The quality of the maps in terms of surveying (survey accuracy, original scale and to what extent the features of interest have been systematically recorded), preservation (deformations and general state), digitization (type of scanning method, resolution and quality) and georeferencing (method employed, resulting RMSE values, number and distribution of GCPs).
- The detection and masking capacity of the detector in terms of counting precision and recall and shape accuracy. Some features will be more easily and unequivocally detected than others, and this limitation must be taken into account when using these data for archaeological analysis and interpretation. Sites themselves are rarely detected, but proxies that can be used to extract information about sites can be documented. Given the variations in map quality discussed above, this information can only be considered an approximation of the true number, size, form and location of the features of interest.
- The possibility of incorporating further datasets, both from other remote sources, such as satellite imagery, and through field‐based ground checking. Given the inexact nature of counts, locations and shapes, the presence of a small percentage of false positives and the difference in accuracy and recording practices between individual surveyors, we argue that the best way to conceive of the results is through a probabilistic framework. This is particularly true of large‐scale approaches where cross‐referencing of information obtained through different methods and sources can be used to weight possible sites. The use of complementary approaches and sources has enormous potential to obtain probabilistic site distribution maps across large areas.
- The range and scale of the map series available. These approaches are most useful when applied to large map series where objects from many maps can be employed to train the different detectors and the time invested in training them will be compensated by their application to several hundred maps. Colonial map series, in particular, show similar symbology and survey approaches, and they extend across very large areas, often spanning several modern countries. These are factors that can make the development of multiple object‐focused detectors worth the time invested in training them, particularly in comparison to manual approaches. The use of these techniques for small areas composed by a few maps is not recommended as it will be difficult to obtain enough training data to develop an efficient detector and the time required to do this may exceed that which would be needed for expert‐led manual detection.

The positive results of this first application of object segmentation using the SoI and French Levant maps opens up the possibility of scaling up our analysis to larger areas covered by these colonial map series. Other large map series, such as those produced by Soviet cartographers across the USSR and parts of Europe and Asia (Davies & Kent, 2017), offer similar potential. We hope that in time, colonial map series can be used for the understanding and protection of cultural heritage and local cultures instead of the direct and indirect exploitation for which they were originally intended.

## CONFLICT OF INTEREST

The authors declare that no conflict of interest exist.

## Data Availability

Though the code used in this paper cannot be shared given the use of a proprietary cloud computing platform to train and run the detection process, readers can access the training data and results obtained for each of the detectors, following this link, which requires a free Picterra's account (https://forms.gle/L8YngAd87eckYSDQA). This proof of concept aimed to test the use of DL approaches to the extraction of features of archaeological interest form historical map series. The paper's results confirm the potential of DL‐based segmentation. Future research will gear towards the development of effective open‐source detectors trained using larger collections of diverse feature types that will significantly improve the results presented in this paper.
